# Human papillomavirus prevalence and genotype distribution landscapes in Shannan City, Tibet Tibetan Autonomous Region, China

**DOI:** 10.1186/s12985-022-01775-5

**Published:** 2022-03-18

**Authors:** Dilu Feng, Sitian Wei, Jun Chen, Zhicheng Yu, Yeshe Lhamo, Hongbo Wang, Xiaowu Zhu

**Affiliations:** 1 Department of Gynecology and Obstetrics, Union Hospital, Tongji Medical College, Huazhong University of Science and Technology, Wuhan, Hubei People’s Republic of China; 2 Department of Gynecology and Obstetrics, Shannan Maternal and Child Health Hospital, Shannan, Tibet People’s Republic of China

**Keywords:** Human papillomavirus, Cervical cancer, Prevalence, Genotype

## Abstract

**Background:**

Data regarding human papillomavirus (HPV) prevalence and genotype distribution are limited in Shannan City, Tibet Tibetan Autonomous Region, China. The purpose of this study is to provide reliable data for guiding women in Shannan City in cervical cancer screening and HPV vaccine innoculation.

**Methods:**

HPV testing was performed on women aged 16–109 years (mean age 44.03 ± 9.25 years) from Shannan City in 2019 and 2020, which was implemented technically by gynecological examination, vaginal discharge smear microscopy, cytology, and HPV detection. The overall prevalence, age-specific prevalence, and genotype distribution were analyzed.

**Results:**

A total of 48,126 women received HPV testing, of which 3929 were detected human papillomavirus. The HPV-positive rate was 8.16% (3929/48,126), and the highest prevalence was in the ≤ 25-year-old age group (12.68%). After the age of 25, the prevalence rate decreased rapidly, and then slowly increased from 7.49% in the 46–55 age group to 9.82% in the ≥ 66 age group, showing a “U-shaped” pattern. The positive prevalence of HPV 16 or 18-only was 1.43%, that of other HPV genotypes except HPV 16 or 18 was 6.39%, and mixed HPV infections including HPV 16 or 18 was 0.34%.

**Conclusions:**

The HPV infection rate in Shannan city is rather low, and the age-specific prevalence of HPV infection presents a “U” curve, suggesting the importance of screening among younger women and the necessity of detection among older women.

## Background

Cervical cancer is one of the common malignant tumors in gynecology. According to the latest global cancer burden data in 2020 released by the international agency for research on cancer (IARC) of the World Health Organization, cervical cancer is the fourth most common female cancer in China and the fourth leading cause of female death [1]. Human papillomavirus (HPV) infection is the main cause of cervical cancer and intraepithelial neoplasia (CIN) [2, 3]. High risk HPV DNA is detected in 99.7% of cervical cancer patients [4], of which HPV 16 and 18 subtypes are the most common causative factors [5]. Prevention of cervical cancer starts with the prevention of HPV infection. Previous studies have shown that the HPV prevalence has obvious regional and age-specific distribution [6, 7]. Understanding the characteristics of HPV infection plays an important role in the prevention and treatment of cervical cancer. In this study, We collected basic information and HPV high-risk subtypes of patients from Shannan Maternal and Child Health Hospital from January 2019 to December 2020, and analyzed the HPV infection landscapes in different and continuous age groups to intervene early in cervical precancerous lesions and cervical cancer, thereby reducing the incidence rate of cervical cancer in Shannan city.

Shannan city is located in Southwest China, with an average altitude of about 3700 m. It is a vast and remote area composed of Tibetan, Han, Hui, and other nationalities. Cervical cancer is still a major public health problem for women living in Shannan city due to the lack of Health Science Popularization for HPV detection and prevention, insufficient local health conditions, scattered population, and ethnic culture. However, data on HPV infection rates in Shannan city are badly limited.

In the past few decades, cervical cancer vaccination has been carried out in more than 130 countries and regions around the world. In China, the HPV vaccine was approved for marketing on July 31, 2017. But so far, in China, especially in Tibet, only a few school-age women have been vaccinated against human papillomavirus-related diseases. So far, there is no large-scale sample study on the prevalence of human papillomavirus genotypes in Tibet.

Here, we examined the HPV infection rate of women in Shannan city and its relationship with age according to the data collected from female patients. These data will provide epidemiological evidence to support the development of a comprehensive and correct cervical cancer prevention program. We also aim to emphasize the need for complete, thorough and detailed data collection to increase our understanding of HPV infection rates.

## Methods

### Study population

A total of 48,126 females aged 16–109 years were enrolled in this study from January 2019 to December 2020. All participants were from Shannan Maternal and Child Health Hospital. Patients visited the hospital for various reasons, including physical examination, vaginitis, abnormal vaginal bleeding, Gynaecological tumors, etc. Clinical information was collected from the patients, and a molecular survey of HPVs was conducted. Eligible women were included in the study after signing an informed consent form.

### Sample collection

Doctors from Wuhan Union Hospital (Wuhan, China) and local clinics were trained in the collection of cervical cell samples for HPV testing. All participants were informed to avoid sex within 24 h before collecting samples. With the help of a vaginal endoscope, the orificium externum isthmus was fully exposed and was scraped through clockwise 5 rotations gently using the HPV detection brush, so that the mucosa and secretions could adhere to the flat sides of the bristles. Then the tips of the cervical brush were put into a vial containing transport medium separately, which were stored at 2–8 °C until HPV DNA extraction and genotyping could be carried out. The HPV genotype detection of samples was completed within 48 h.

### DNA extraction, PCR amplification, and HPV genotyping

HPV DNA was extracted from cervical samples using a viral nucleic acid extraction kit (Bioperfectus Technologies Co., Ltd., Taizhou, Jiangsu, China). In short, cervical cells were first digested with lysis buffer. Then, DNA was extracted by magnetic bead method, and DNA was washed and purified from these particles using an automatic nucleic acid extractor (Bioperfectus Technologies Co., Ltd., Taizhou, Jiangsu, China).

The diagnostic kit for high-risk human papillomavirus (HPV) (fluorescent PCR) (Bioperfectus Technologies Co., Ltd., Taizhou, Jiangsu, China) was used for HPV DNA amplification and genotyping. The kit was used for the qualitative detection of 18 high-risk HPV (HPV16, 18, 31, 33, 35, 39, 45, 51, 52, 56, 58.59, 66, 68, 73.53, 82, and 26) nucleic acids in women's cervical exfoliated cells in vitro, HPV16 and HPV18 were specifically distinguished, but the other 16 high-risk types were not specifically classified. This kit used real-time fluorescent PCR technology to label HPV16, HPV18, other 16 high-risk HPVs, and internal standard (β-globin) with FAM, VIC, ROX, and CY5 fluorescein respectively, so it could realize the specific distinction between HPV16 and HPV18. Primers were designed on 18 high-risk HPV-specific genes. The target genes designed by HPV primer probes were L1, L2, and E1 genes, and the length of each type of amplicon was no more than 200 bp. 5 μl DNA was added to format a 25 μl PCR reaction system. SLAN-96P real-time fluorescence quantitative PCR instrument (Hongshi Medical Technology Co., Ltd., Shanghai, China) was used for PCR amplification detection. The PCR reaction began with denaturation at 95 °C for 10 min, followed by 45 cycles, denaturation at 95 °C for 15 s, and annealing and extension at 58 °C for 50 s. Fluorescence was collected during the annealing extension phase. According to the verification with the comparative reagent clinical trial and the third-party reagent, the receiver operating characteristic curve (ROC) was used to determine that the cutoff value of HPV16, HPV18, and other 16 high-risk types was 35.4, 34.6, and 33.6 respectively.

Quality control was carried out throughout the whole experiment, including PCR amplification and hybridization using the positive and negative controls provided by the kit. Experimental procedures were all followed by the manufacturer's manual.

### Statistical analysis

Analyses were done with SPSS (version 25.0) and Excel (version 2004). Descriptive statistical analysis was performed on the distribution of HPV genotypes using indicators such as frequency and prevalence. For comparisons among different age groups, the categorical variables were compared by using the Chi-square test. All the reported *P* values were made based on two-sided tests with a significance level of 0.05.

## Results

### Prevalence and genotype distribution of human papillomavirus

From January 2019 to December 2020, a total of 48,126 human papillomavirus genotype samples were collected and detected in Shannan Maternal and Child Health Hospital. The subjects were aged 16–109 years, and the mean age was 44.03 ± 9.25 years. The genotype test showed that 3929 samples were HPV positive, so the overall prevalence of all types of human papillomavirus in the study population was 8.16%.

The details of genotypes distribution were shown in Tables 1 and 2. Notably, HPV 16-only infection rate was 0.52% (250/48,126). Correspondingly, HPV 16-total was 0.70% (339/48,126). HPV 18-only infection rate was 0.91% (440/48,126) and HPV 18-total was 1.09% (526/48,126), respectively. In addition, the HPV infection rate of other genotypes was 6.73% (3239/48,126), accounting for 82.44% (3239/3929) of all HPV-positive samples.

**Table 1:** Frequency and prevalence of genotypes of HPV among women

Genotype	Positive samples (n)	Proportion among 48,126 samples (%)	Proportion among 3929 HPV-positive samples (%)
16	250	0.52	6.36
18	440	0.91	11.20
Others*	3077	6.39	78.32
16 + others	76	0.16	1.93
18 + others	73	0.15	1.86
16 + 18 + others	13	0.03	0.33

^*^Other genotypes of HPV was HPV 31, 33, 35, 39, 45, 51, 52, 56, 58.59, 66, 68, 73.53, 82, and 26

**Table 2:** Frequency and prevalence of HPV16, 18 and other genotypes among women

Genotype	Positive samples (n)	Proportion among 48,126 samples (%)	Proportion among 3929 HPV-positive samples (%)
16	339	0.70	8.63
18	526	1.09	13.39
Others*	3239	6.73	82.44

^*^Other genotypes of HPV was HPV 31, 33, 35, 39, 45, 51, 52, 56, 58.59, 66, 68, 73.53, 82, and 26

### Prevalence of HPV grouped by age

Overall, 48,126 women (aged 16–109 years) were included in this study. All the participants were divided into six groups ranging from ≤ 25 years, 26–35 years, 36–45 years, 46–55 years, 56–66 years, and ≥ 66 years. There were significant differences in HPV infection rates among the six age groups (*P* < 0.05). Relevant data could be seen in Table 3.

**Table 3:** Prevalence of HPV grouped by age groups

Age group (years)	Mean age (years)	Total samples (n)	Positive samples (n)	Proportion among total samples (%)
≤ 25	23.32 ± 1.80	891	113	12.68
26–35	31.99 ± 2.79	8289	726	8.76
36–45	40.35 ± 2.85	17,640	1415	8.02
46–55	50.15 ± 2.80	15,438	1157	7.49
56–65	58.76 ± 2.35	5705	502	8.80
≥ 66	69.57 ± 6.31	163	16	9.82
Total	44.03 ± 9.25	48,126	3929	8.16
χ^2^	41.542			
*P*	< 0.05			

**Fig. 1:**
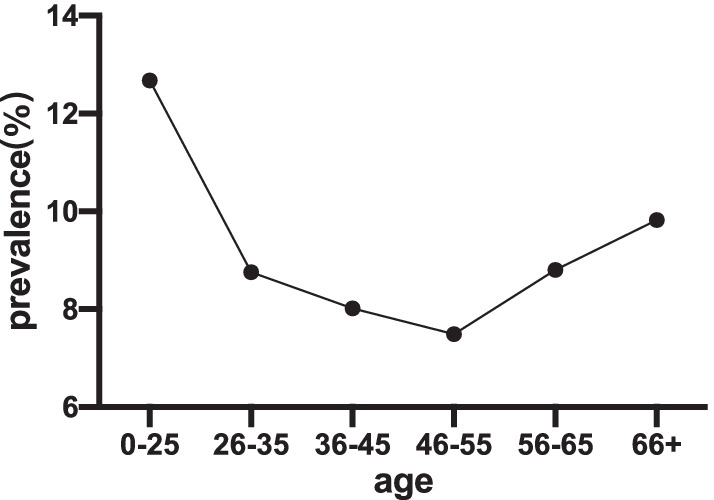
Prevalence of HPV infection in different age intervals. The highest HPV infection rates was in women aged ≤ 25 years (12.68%), and the lowest was in the 46–55 age group (7.49%). The overall performance was a “U” shape

The distribution of HPV infection rates in specific age groups presented a “U” curve. The first peak appeared in the youngest women (12.68%). However, the HPV infection rate decreased sharply after the first peak, and reached the bottom in the 46–55 age group (7.49%). On the contrary, it increased slowly, and finally reached the second peak at 9.82% in the oldest women group (Fig. 1).

### Region-specific prevalence of HPV infection

The prevalence of HPV infection in Shannan city and in 19 different areas of China was compared separately (Table 4). There were significant differences in HPV prevalence among these areas (*P* < 0.05). The positive rate of HPV obtained in our study (Shannan City) was significantly different from those in other areas (*P* < 0.05). Moreover, the highest rate of HPV infection was in Henan Province (38.10%, 1536/4033) [8], whereas the lowest area was in Shannan City (8.16%, 3929/48,126). These results indicated that HPV infections were significantly region-specific.

**Table 4:** Comparison of HPV infection rates in different areas in China

Regions	Total samples (n)	Positive samples (n)	Prevalence (%)	*P*-value
Shannan City	48,126	3929	8.16	–
Beijing City [9]	29,436	3586	12.18	< 0.001
Shanghai City [10]	23,724	3816	16.08	< 0.001
Wuhan City [11]	13,775	2436	17.68	< 0.001
Xinjiang Province [12]	37,722	5287	14.02	< 0.001
Jiangxi Province [13]	71,435	16,065	22.49	< 0.001
Sichuan Province [14]	14,185	3382	23.84	< 0.001
Yunnan Province [15]	17,898	3681	12.94	< 0.001
Zhejiang Province [11]	77,443	17,270	22.30	< 0.001
Guangdong Province [16]	33,328	3526	10.58	< 0.001
Jiangsu Province [17]	62,317	16,775	26.92	< 0.001
Shaanxi Province [18]	17,341	4559	26.30	< 0.001
Shandong Province [19]	94,489	26,839	28.40	< 0.001
Henan Province [8]	4033	1536	38.10	< 0.001
Shanxi Province [20]	7640	1441	18.86	< 0.001
Liuzhou City [21]	2300	522	22.70	< 0.001
Jilin Province [16]	20,648	7095	34.40	< 0.001
Heilongjiang Province [22]	18,522	5011	27.10	< 0.001
Liaoning Province [23]	6479	667	10.30	< 0.001
Inner Mongolia [24]	2345	844	36.00	< 0.001
χ^2^	18,795.358			
*P*	< 0.05			

## Discussion

HPV screening is very important for the prevention and detection of cervical cancer. However, cervical cancer screening started late in China, and there is still a big gap between the current promotion and developed countries. In addition, the data of HPV infection rate and genotype distribution in different regions are not comprehensive, especially in the remote areas in Western and Northern China. There is a lack of relevant data to guide the health education of cervical cancer. Therefore, collecting and analyzing the epidemiological evidence of local HPV infection can provide a reliable scientific basis for the prevention, treatment, and elimination of human papillomavirus infection-related diseases.

Shannan city of Tibet Tibetan Autonomous Region was located in Northwest China. Its economy was underdeveloped, and the prevention and screening of cervical cancer were also quite backward. Therefore, improving the screening of HPV in Tibet was particularly important for the primary prevention of cervical cancer in Tibet. This is not only conducive to women's health but also can save a lot of medical expenses for the country. Studying the prevalence and genotype distribution of human papillomavirus in different regions and periods is highly important for cervical cancer screening and evaluating the effectiveness of the human papillomavirus vaccine for women. For the last 20 years, the world has made great efforts to generate epidemiological data on cervical HPV-DNA. In China, although certain studies have been performed to assess the prevalence and incidence rate of human papillomavirus genotypes in Tibet, they are based on small samples [25, 26]. Our study is the first large-scale sample study in Tibet.

80% of the high-risk HPV DNA detected in patients with cervical cancer were HPV16, 18, 45, and 31 [4]. Therefore, in this study, we mainly focused on the infection rates of HPV16 and 18. It could be seen that HPV16 and 18 positive accounted for 8.63% and 13.39% of 3929 HPV positive samples respectively. By now, all three existing vaccines could prevent HPV16 and 18 [27]. Therefore, for women in Shannan City, Tibet, no matter how much valent vaccine they chose, they could meet their needs for the prevention of cervical cancer. As expected in this study, clarifying the distribution of HPV genotype infection rate in specific areas was conducive to HPV vaccine development and clinical application.

Due to the differences in regional, population, living environment and lifestyle, and human papillomavirus DNA test methods, the reported results of global human papillomavirus distribution vary from study to study. A meta-analysis [28] of a total of 1,016,719 screening people included in 194 studies around the world showed that the adjusted infection rate of HPV in the global population with normal cytology was 11.7%, of which the infection rate was the highest in sub-Saharan Africa (24.0%), Eastern Europe (21.4%) and Latin America (16.1%) and the lowest in Western Asia (1.7%). According to research, the overall prevalence of human papillomavirus infection in China is 15.54% [29]. In our study, the rate of human papillomavirus infection among women in Shannan City, Tibet was 8.16%, lower than the global average, lower than the Chinese average, and lower than many other cities or regions in China. Some researchers speculated that the variability of HPV prevalence in China was due to China's large population and territory [29]. At the same time, the level of economic development and population migration also led to the differences in the distribution landscapes of human papillomavirus among regions. As shown in Table 4, both Beijing and Shanghai were economically developed cities, but the HPV infection rate varied greatly [9, 10]. The reason may be that Beijing, as China's political, cultural, and economic center, had good medical conditions and protection strategies. Otherwiese, Shanghai's economic development level was also very high, but due to a large number of foreigners and migrant population, the city was expanding and the population composition was complex, resulting in a high rate of human papillomavirus infection. In Shannan City, although the economy and medical treatment were relatively backward, the HPV infection rate was the lowest. The reason behind it may be the simple folk customs, the majority of the people who believe in Tibetan Buddhism and the conservative traditional concept of sex, which was similar to Xinjiang Provence. Another study on the HPV infection rate in Tibet which was 9.19% (279/3036), which supports this conclusion [25].

Information on the distribution of human papillomavirus infection in different age groups is extremely important for the design of human papillomavirus preventive vaccines in specific age groups [30]. What many studies have in common is that the first peak occurs in the younger age group (just after the beginning of sexual relations). In some areas, the second peak can be observed at the age of > 45 or > 55 or > 65, while in some other areas, no second peak can be observed. In conclusion, age-specific HPV distribution is either shown as a bimodal curve (including "U" curve) [31, 32] or a left inclined unimodal distribution [26]. In this study, age-specific HPV distribution showed a "U" curve. The first peak of human papillomavirus infection occurred in the age group ≤ 25 years old (12.68%), then decreased gradually, reached the lowest in the age group 46–55 years old, and then increased gradually. The reason for this trend may be that young women were sensitive to human papillomavirus soon after sexual activity due to immature immune protection [33]. With the stimulation of immune response, a large part of primary HPV infection would be temporary and would be cleared spontaneously [34], so the HPV infection rate decreased gradually. The immune ability of elderly women decreased with age, especially in the premenopausal and postmenopausal women, which resulted their ability to eliminate previous and new HPV infections being weakened. Furthermore, as past (latent) infections reappeared, both factors led to a higher HPV infection rate of elderly women [35]. Based on these findings, the earlier young women were vaccinated with HPV vaccine, the higher the antibody titer and the better the protection [36]. Once HPV infection was detected, HPV viral load should be continuously monitored and cervical biopsy should be performed regularly.

Although a variety of studies provided large-scale information on the recent HPV prevalence and genotype distribution in Shannan City, Tibet, China, there were still some limitations. First, most cervical screening tests received by patients did not carry out detailed HPV typing, nor did they be combined with cytology. Women included in some studies were unable to obtain cervical cytological or histological results. Therefore, it is impossible for doctors or researchers to associate HPV infection and genotype distribution with different cervical abnormalities. Secondly, the collection of personal information of patients was not complete. Tibet had a high altitude and a wide area. There was a variety of distinctive information about the nationality, living habits, reproductive history, climatic conditions cultural, and other backgrounds of the population in this area. Unfortunately, it was not recorded in this study so that we couldn’t specify the impact of these different backgrounds on the rate of HPV infection. In addition, some studies had shown that human papillomavirus infection may lead not only to cervical cancer but also to oropharyngeal cancer and head and neck cancer [37]. Therefore, it was suggested to also focus on the relationship between HPV infection and oropharyngeal cancer, and head and neck cancer in Tibetan women in future research. In addition, obtaining data on human papillomavirus infection in Tibetan men could be regarded as a potential direction for future research.

After all, our study revealed the HPV infection rate and genotype distribution of women from Shannan City, Tibet, China. This information may provide guidance and suggestions for the prevention of cervical cancer for women in this area. According to the current results, young women should be vaccinated with HPV vaccine as soon as possible, and elderly women should focus on crevice screening.

## Conclusion

In summary, this study analyzed the prevalence of HPV in women and its difference with age distribution characteristics in Shannan City, Tibet from 2019 to 2020. Moreover, the differences of HPV infection rate between Shannan city and other regions in China were also compared. The results in our study will provide helpful information for cervical cancer screening and human papillomavirus vaccination in women in Shannan City, Tibet.

## Data Availability

The data was collected from Shannan Maternal and Child Health Hospital. We thank them for their generous help.

## Abbreviations

HPV: Human papillomavirus
CIN: Cervical intraepithelial neoplasia
ROC: Receiver operating characteristic curve
